# Systematic Review of Compound Danshen Dropping Pill: A Chinese Patent Medicine for Acute Myocardial Infarction

**DOI:** 10.1155/2013/808076

**Published:** 2013-06-17

**Authors:** Jing Luo, Hao Xu, Keji Chen

**Affiliations:** ^1^Graduate School, Beijing University of Chinese Medicine, Beijing 100029, China; ^2^Cardiovascular Diseases Center, Xiyuan Hospital, China Academy of Chinese Medical Sciences, Beijing 100091, China

## Abstract

*Objective*. This paper systematically evaluated the efficacy and safety of compound Danshen dropping pill (CDDP) in patients with acute myocardial infarction (AMI). *Methods*. Randomized controlled trials (RCTs), comparing CDDP with no intervention, placebo, or conventional western medicine, were retrieved. Data extraction and analyses were conducted in accordance with the Cochrane standards. We assessed risk of bias for each included study and evaluated the strength of evidence on prespecified outcomes. *Results*. Seven RCTs enrolling 1215 patients were included. CDDP was associated with statistically significant reductions in the risk of cardiac death and heart failure compared with no intervention based on conventional therapy for AMI. In addition, CDDP was associated with improvement of quality of life and impaired left ventricular ejection fraction. Nevertheless, the safety of CDDP was unproven for the limited data. The quality of evidence for each outcome in the main comparison (CDDP versus no intervention) was “low” or “moderate.” *Conclusion*. CDDP showed some potential benefits for AMI patients, such as the reductions of cardiac death and heart failure. However, the overall quality of evidence was poor, and the safety of CDDP for AMI patients was not confirmed. More evidence from high quality RCTs is warranted to support the use of CDDP for AMI patients.

## 1. Introduction

Acute myocardial infarction (AMI) is a serious type of coronary heart disease (CHD) and a major cause of death worldwide with an estimated annual incidence rate of seven million people [1]. As a result of coronary artery thrombotic occlusion from plaques rupture or erosion, AMI usually leads to death if complicated by severe heart failure, malignant ventricular arrhythmia, or cardiac rupture [1, 2]. Despite the application of percutaneous coronary intervention (PCI) and conventional western medicine, AMI patients remain at certain risk of in-hospital death and complications as well as recurrent acute cardiovascular events [2–4]. With more and more clinicians successfully applied traditional Chinese medicine (TCM) in CHD prevention and treatment based on conventional therapy, the effects of TCM for CHD have drawn more and more attention [5–8].

Compound Danshen dropping pill (CDDP, also known as the “Dantonic Pill”), a Chinese oral patent medicine, has been widely used for cardiovascular diseases, including AMI, in China and some Asia countries. The phase II clinical trial of CDDP to treat chronic stable angina (http://clinicaltrials.gov/, NCT00797953) had been completed in the United States in 2010. Moreover, this drug has been approved by the Australian Therapeutic Goods Administration for use and is widely available in Australia [9]. CDDP consists of three compositions, namely, *Radix Salviae Miltiorrhizae*, *Radix Notoginseng*, and *Borneolum Syntheticum*. These compositions and their pharmacological actions [10–15] are listed in Table 1 with common, pinyin, and Latin names. Previous pharmacologic studies and randomized clinical trials have indicated the potential benefit of CDDP for patients with AMI [16–21]. Recent systematic reviews [22–24] also revealed potential benefits of CDDP for angina pectoris. The efficacy and safety of CDDP for AMI, however, have not been systematically evaluated. The aim of this study was to assess the efficacy and safety of CDDP on the treatment of AMI patients.

## 2. Methods

### 2.1. Inclusion and Exclusion Criteria

Randomized controlled trials (RCTs) comparing CDDP with no intervention, placebo, or conventional western medicine were sought regardless of their publication status. Participants of any gender, age, or ethnic origin with AMI meeting with one of the past or current definitions of AMI [25–29] were included. Those without description of diagnostic criteria but stated patients with definite AMI were also considered. Quasi-randomized trials and animal experiments were excluded. Trials with CDDP as adjunctive therapy or with duration less than four weeks were also excluded.

Primary outcomes consisted of all-cause mortality, cardiac mortality, recurrent myocardial infarction (RMI), and revascularization, including PCI and coronary artery bypass graft (CABG). Secondary outcomes included heart failure, readmission, left ventricular ejection fraction (LVEF), recurrent angina, adverse events and health-related quality of life measured by a validated tool.

### 2.2. Source of Literature and Search Strategy

#### 2.2.1. Electronic Searches

We searched the following databases up to October 2012 for the identification of RCTs both published and unpublished: Pubmed, The Cochrane Library, Chinese Biomedical Database (CBM), Chinese VIP Information (VIP), China National Knowledge Infrastructure (CNKI), Wanfang Databases, China Proceedings of Conference Full-text Database (CPCD), Chinese Doctoral Dissertations Full-text Database (CDFD), and Chinese Master's Theses Full-text Database (CMFD). Search strategy in Table 2 was used in The Cochrane Library and adapted appropriately for other databases.

In addition, we searched databases of ongoing trials: ClinicalTrials.gov (http://clinicaltrials.gov/) and Current Controlled Trials (http://www.controlled-trials.com/).

#### 2.2.2. Additional Searches

We also searched the reference lists of studies included in this systematic review and of other relevant reviews to identify missing relevant articles.

### 2.3. Study Identification and Data Extraction

Two authors (Jing Luo, Hao Xu) independently screened the titles and abstracts of references for potentially relevant RCTs. Full texts of potentially eligible articles were retrieved for further identification according to the inclusion and exclusion criteria. Any disagreement was resolved by consensus.

Two authors (Jing Luo, Hao Xu) independently extracted data using a preset data extraction form. Characteristics of RCTs including methods, participants, interventions, comparisons, and outcomes were extracted. We obtained missing information from the original authors whenever possible and resolved any disagreement through discussion or consulting the third author (Keji Chen).

### 2.4. Assessment of Risk of Bias and Quality of Evidence

Two authors (Jing Luo, Hao Xu) independently assessed the methodological quality of each of the included studies using the Cochrane “risk of bias” criteria [30], which covers the following items: random sequence generation, allocation concealment, blinding of participants and personnel, blinding of outcome assessment, incomplete outcome data, selective reporting, and other bias. Disagreements were resolved by consensus. For each item, a low risk was considered when we judged a “Yes,” conversely, a “No” for a high risk, and otherwise for an unclear risk.

We also evaluated the quality of evidence of each outcome using the Grading of Recommendations Assessment, Development and Evaluation (GRADE) approach [31], as recommended by the Cochrane Collaboration. Patient important outcomes in the main comparison were judged across five factors: limitations in study design and execution, inconsistency of results, indirectness of evidence, imprecision, and publication bias. Accordingly, we graded the quality of evidence in this review as very low, low, moderate, or high.

### 2.5. Data Analysis

We used RevMan 5.1 software for data analyses. Studies were stratified by the different types of comparisons. We performed intention-to-treat analysis (ITT) for dichotomous data and presented outcome data as risk ratio (RR) with corresponding 95% confidence interval (CI). We calculated mean difference (MD) with its 95% CI for continuous outcomes. Fixed effect model was used to analyze data with low heterogeneity (*I* ^2^ ≤ 50%); random effects model was applied if heterogeneity is significant (50% < *I* ^2^ < 75%). Results were not pooled for data with high heterogeneity (*I* ^2^ ≥ 75%) [32], in which case we explored potential causes of heterogeneity by conducting subgroup analyses based on the characteristics of intervention (dosage, duration) and the types of conventional therapy (PCI versus thrombolysis). We also performed sensitivity analyses on studies with lower methodological quality, in order to investigate whether the inclusion of such studies altered the conclusion of the meta-analysis. Possible publication bias was checked using funnel plots when the number of included studies of any particular outcome is greater than eight.

## 3. Results

### 3.1. Study Identification

**Figure 1:**
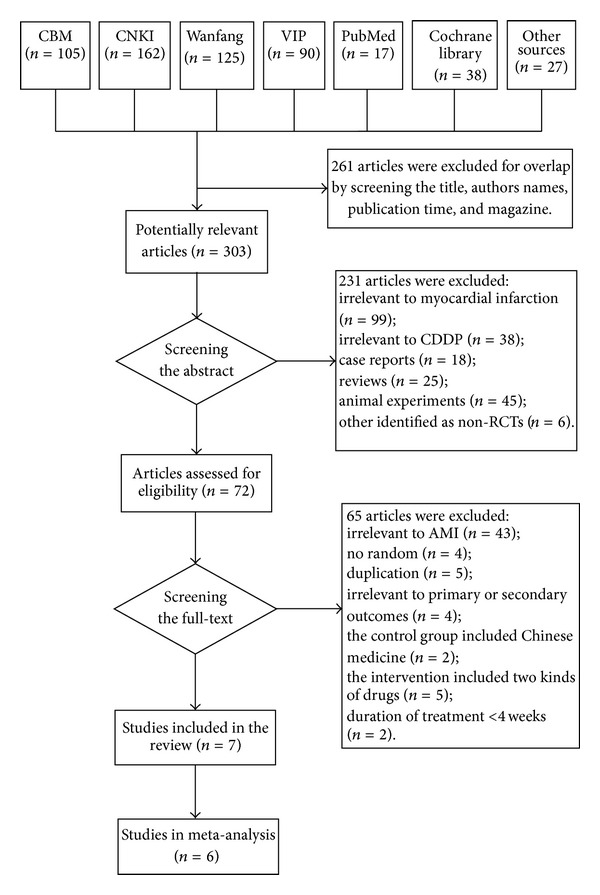
Flow chart of study search and identification.

A total of 564 references were found according to search strategy, of which 261 were excluded for duplicates among databases. After screening the abstract, we excluded 231 articles. 72 potentially eligible studies were retrieved for further identification, of which 65 were excluded because they did not meet the prespecified inclusion criteria described in the methods. At last, seven eligible RCTs [19–21, 33–36] were included. No ongoing trial was found. Please refer to Figure 1 for a more detailed illustration of the data screening process.

### 3.2. Description of Included Studies

The characteristics of the included seven studies [19–21, 33–36] are summarized in Table 3. Each of the studies was conducted in China. One postgraduate dissertation [35] was unpublished in 2010, and the others were published from 2006 to 2011. One study [19] was of multicenter design, but the others were of single centre trials.

The number of participants in the individual study ranged from 45 to 500, with a total of 1215 in this review (583 in intervention groups and 632 in control groups). There were 863 males and 352 females included in the review, with mean age, where given [19, 20, 34–36], ranging from 52 to 66 years. All of the participants were diagnosed with AMI by different diagnostic criteria: two studies [20, 21] used the WHO diagnostic criteria; one study [33] used ACC/AHA diagnostic criteria; four studies [19, 34–36] without specified diagnostic criteria but mentioned “patients with AMI were eligible to include.” Two studies [19, 35] only included patients with ST-elevation myocardial infarction (STEMI), one study excluded AMI without Q wave [21], and the others did not introduce the types of AMI (four studies) [20, 33, 34, 36].

All participants in the intervention groups were treated with CDDP, 10 pills three times a day (tid) orally based on conventional therapy since the day of diagnosis [19, 20, 33–36]. Only one study [21] began the CDDP treatment four to five weeks later after diagnosis and changed the dosage from 10 pills tid to five pills tid after 60 days of treatment. The duration of treatment was mainly as same as the length of follow up, ranging from four weeks to 12 months. One study [21] was designed as three groups with two comparisons including CDDP versus no intervention and CDDP versus propranolol. Six studies consisted of two groups (one study [36] compared CDDP with placebo and the others [19, 20, 33–35] focused on CDDP compared with no intervention). In total, there were three comparisons in the review: (1) CDDP plus conventional therapy versus conventional therapy (six studies) [19–21, 33–35]; (2) CDDP plus conventional therapy versus placebo plus conventional therapy (one study) [36]; (3) CDDP plus conventional therapy versus propranolol plus conventional therapy (one study) [21].

Five studies [19–21, 35, 36] reported mortality including all-cause mortality (four studies) [19–21, 35] and cardiac mortality (three studies) [21, 35, 36]. Two studies provided numerical information on RMI [21, 36], but the data could not be pooled for the different comparisons. Four studies [19–21, 36] reported heart failure. Three studies [19, 20, 36] provided the number of patients having recurrent angina. Besides the incidence of readmission and adverse events (narrative introduction), one study [36] also assessed the QOL by questionnaire score, and the questionnaire was designed referring to Treatment of Mild Hypertension Study (TOMHS) and Medical Outcomes Study 36-Item Short- Form Health Survey (SF-36). Five studies [19, 21, 33, 34, 36] assessed the LVEF with the aim of evaluating the heart function. None of the included studies mentioned revascularization.

### 3.3. Quality of Included Studies

#### 3.3.1. Risk of Bias in Included Studies

**Figure 2:**
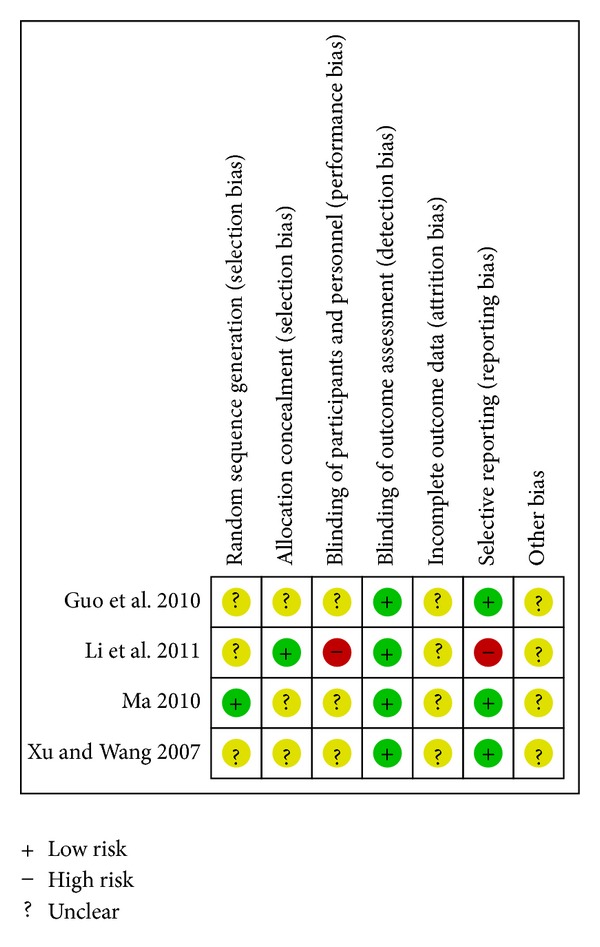
Risk of bias summary—all-cause mortality.

**Figure 3:**
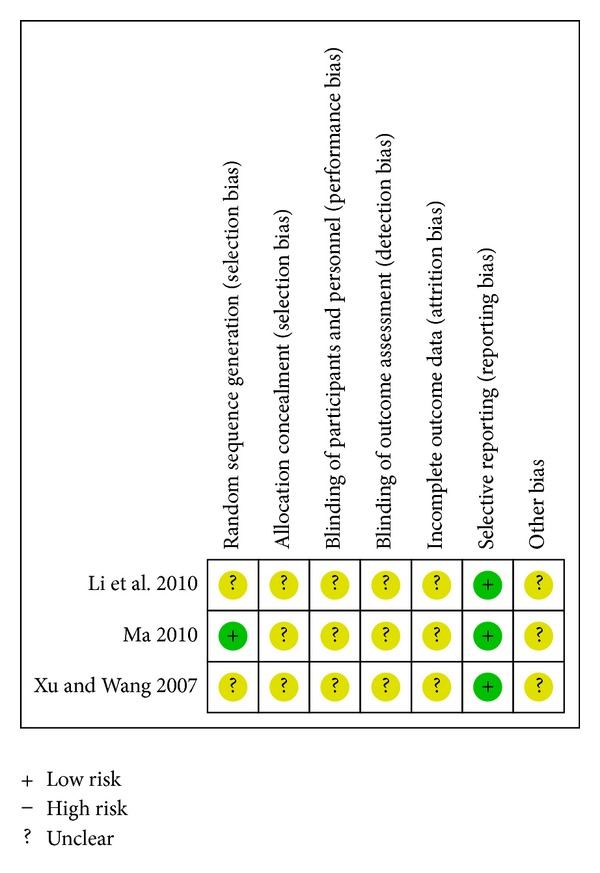
Risk of bias summary—cardiac mortality.

**Figure 4:**
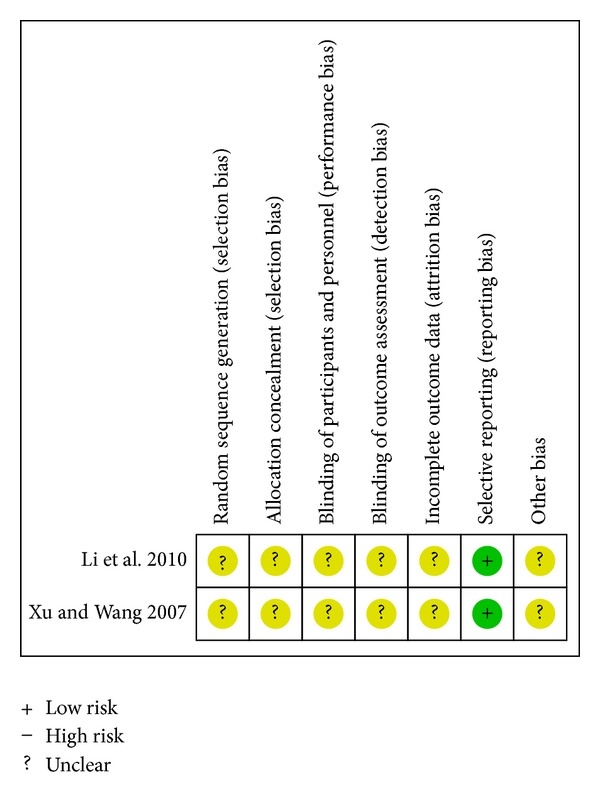
Risk of bias summary—recurrent myocardial infarction.

**Figure 5:**
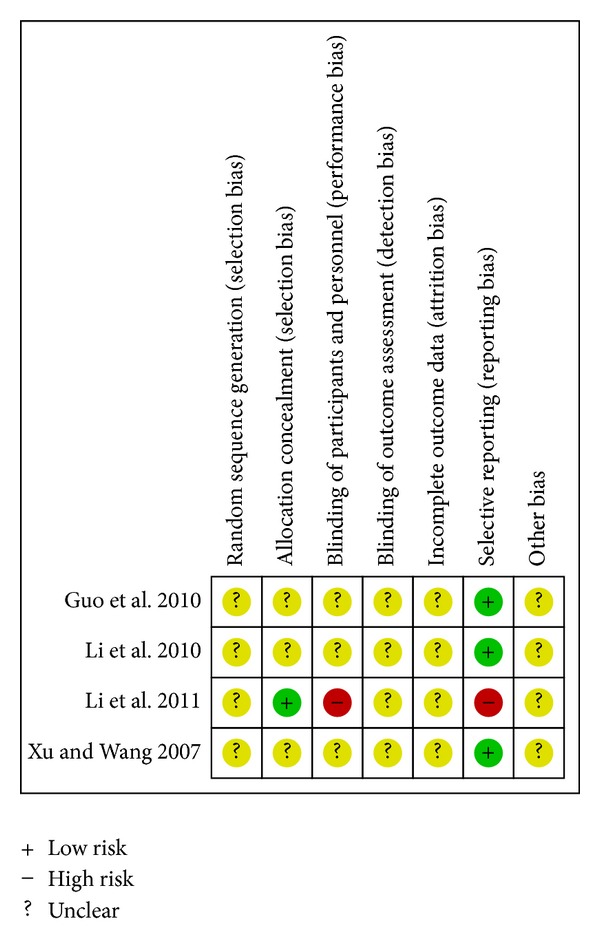
Risk of bias summary—heart failure.

**Figure 6:**
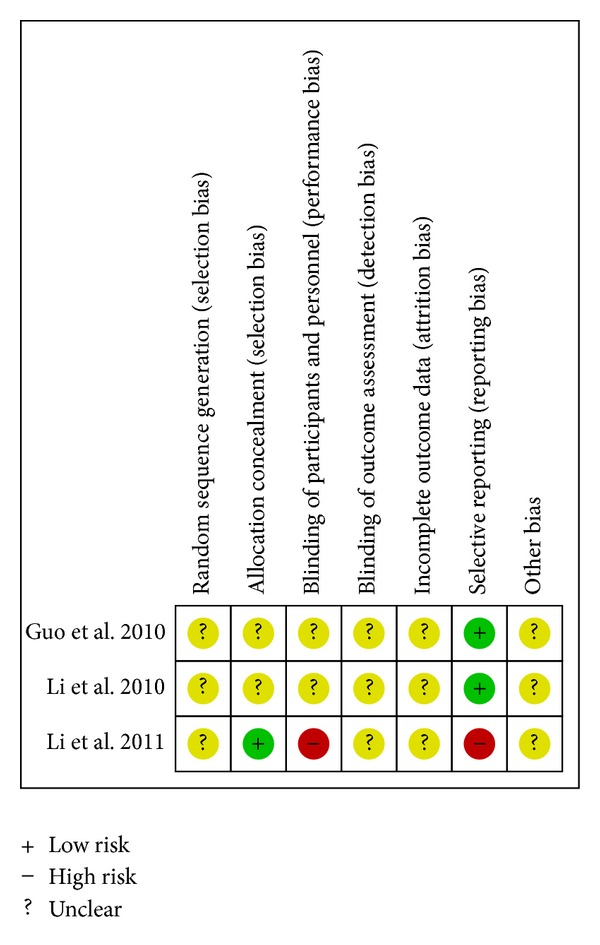
Risk of bias summary—recurrent angina.

**Figure 7:**
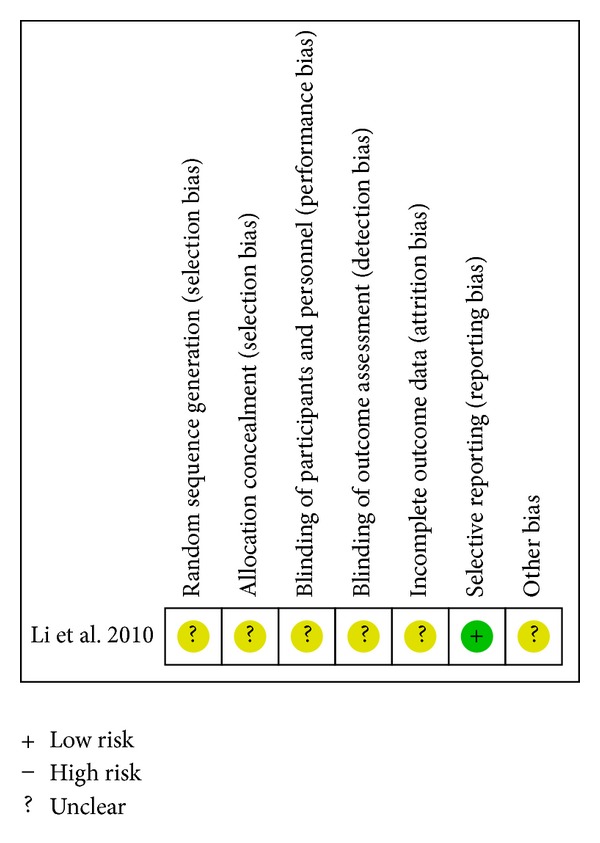
Risk of bias summary—readmission.

**Figure 8:**
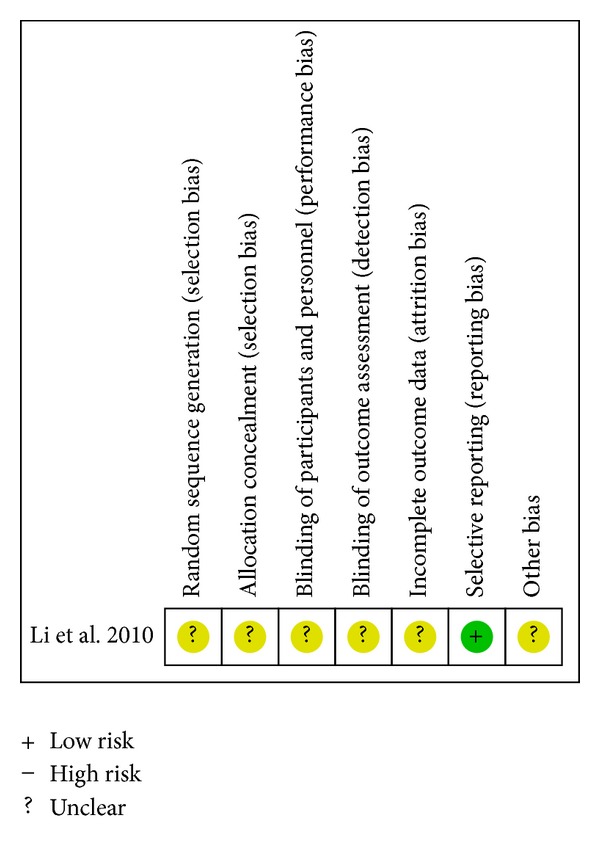
Risk of bias summary—QOL.

**Figure 9:**
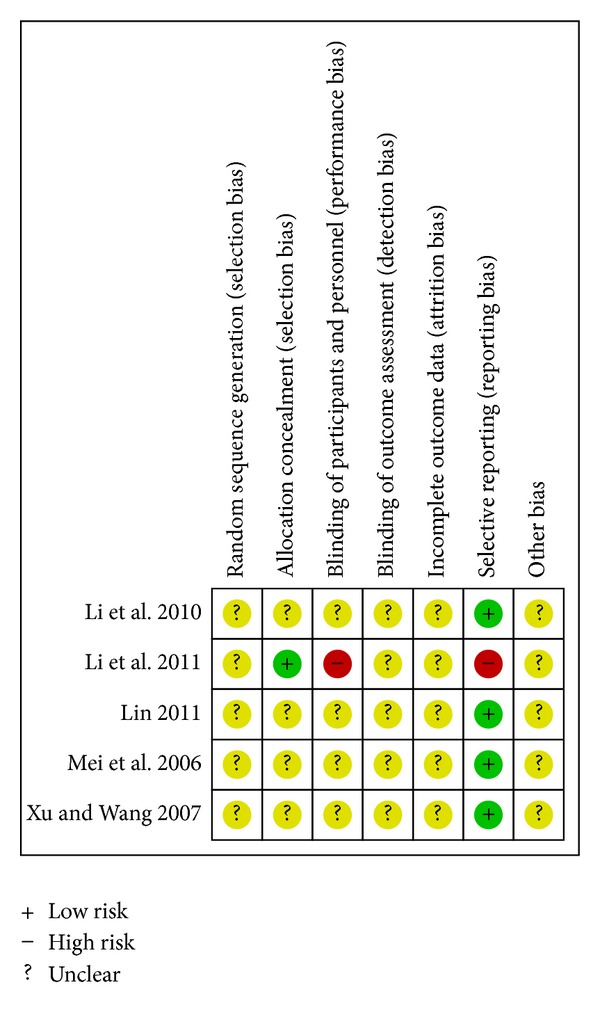
Risk of bias summary—LVEF.

**Figure 10:**
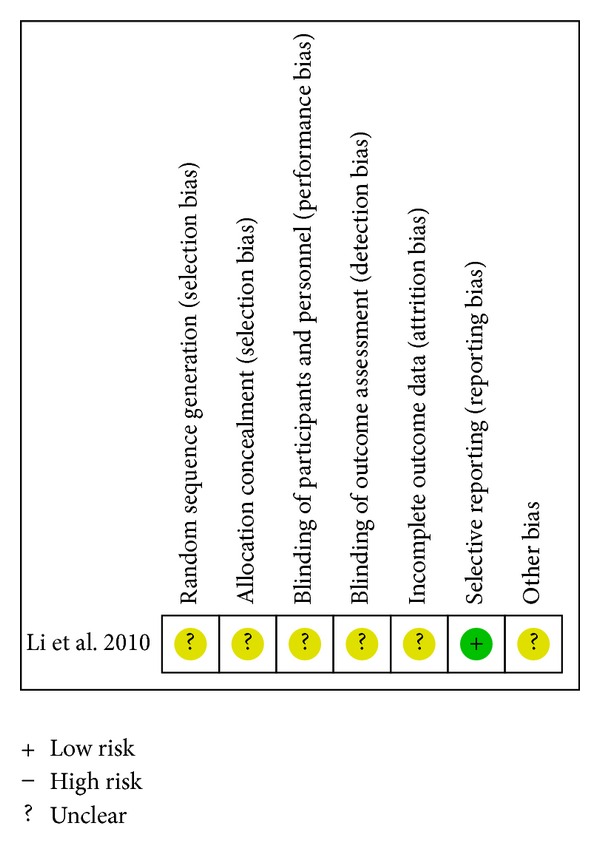
Risk of bias summary—adverse events.

Risk of bias summaries for each outcome in the included RCTs at the study level are presented in Figures 2 –10. No study was felt to have a low risk of bias. Of the seven studies, one [35] introduced the random sequence being generated from a random number table, and the others just mentioned “patients were randomly allocated” without the method of randomization. Only one study [19] reported allocation concealment. None of the studies described blinding of participants and personnel although one study [36] used placebo. All of the studies did not report blinding of outcome assessment. Neither withdrawals nor losses to follow up were reported in the studies. One study [19] had incomplete outcome data. Five studies [20, 21, 33, 35, 36] reported the comparability of the baseline among groups, but four of them did not provide baseline data [20, 21, 33, 36]. The multicenter study [19], with other similar baselines, reported that the rate of diabetes patients in the intervention group was higher than the control group. In addition, no study mentioned prior sample size estimation or ITT analysis for any outcome.

After we contacted with the original authors by telephone and email, only one author [19] told us that there was no blinding of participants or personnel in their study, and the randomization was designed by public health statistics teaching and research section of Tianjin Medical University; he did not know any other details. In fact, due to a number of unsuccessful contacts and some unclear or unavailable replies, most of our questions were not resolved.

#### 3.3.2. Quality of Evidence in Included Studies

The quality of evidence for each outcome in the main comparison (CDDP versus no intervention) was ranged from “low” to “moderate” (Table 4). Quality assessment of the evidence in accordance with the GRADE approach showed some limitations of the study design and execution, inconsistency, indirectness, and imprecision. Due to the low number of included studies for each outcome, we could not create funnel plots to detect publication bias. For each outcome, there were one or two serious limitations among the five factors. For example, because of the serious risk of bias and imprecision for all-cause mortality in the main comparison, we downgraded the quality rating by two levels, thus the quality of evidence for this outcome was low. The quality of evidence was moderate for cardiac mortality and heart failure, low for all-cause mortality, RMI, recurrent angina, and LVEF.

### 3.4. Effect of Interventions (Table 5 to Table 7)

#### 3.4.1. All-Cause Mortality (Table 5)

Four studies [19–21, 35] reported all-cause mortality in two different comparisons. Meta-analysis of the four studies showed no statistically significant difference in the risk of all-cause death between CDDP and no intervention (RR 0.65; 95%CI 0.37 to 1.14; *n* = 945). Sensitive analysis, excluding the lower quality study [19], found that CDDP was associated with a statistically significant reduction in the risk of all-cause death compared with no intervention without heterogeneity (RR 0.51; 95%CI 0.27 to 0.98; three studies, *n* = 445; *I* ^2^ = 0%) [20, 21, 35]. A single study reported that there was no statistical difference in reducing all-cause mortality between CDDP and propranolol on the basis of conventional therapy (RR 0.65; 95%CI 0.16 to 2.63; *n* = 138) [21]. The associated risk of bias is presented in Figure 2. The quality of evidence in the main comparison (CDDP versus no intervention) was low (Table 4).

#### 3.4.2. Cardiac Mortality (Table 5)

Three studies [21, 35, 36] assessed cardiac mortality in three different comparisons. Meta-analysis of two studies [21, 35] showed that CDDP was associated with a statistically significant reduction in the risk of cardiac death compared with no intervention without heterogeneity (RR 0.43; 95%CI 0.20 to 0.95; *n* = 309; *I* ^2^ = 0%). Compared with placebo on the basis of conventional therapy, CDDP had no statistically significant advantage in reducing cardiac mortality (RR 0.50; 95%CI 0.03 to 7.60; one study, *n* = 63) [36]. A single study reported a similar result between CDDP and propranolol (RR 0.81; 95%CI 0.17 to 3.76; *n* = 138) [21].  Figure 3 presents the associated risk of bias. The quality of evidence in the main comparison (CDDP versus no intervention) was moderate (Table 4).

#### 3.4.3. Recurrent Myocardial Infarction (Table 5)

Two studies [21, 36] reported RMI in three different comparisons. None of the comparisons, however, presented a statistically significant difference in the risk of RMI: CDDP versus no intervention (RR 0.30; 95%CI 0.07 to 1.38; one study, *n* = 146) [21]; CDDP versus placebo (RR 0.50; 95%CI 0.11 to 2.27; one study, *n* = 63) [36]; CDDP versus propranolol (RR 0.73; 95%CI 0.13 to 4.22; one study, *n* = 138) [21]. The associated risk of bias is presented in Figure 4. The quality of evidence in the main comparison (CDDP versus no intervention) was low (Table 4).

#### 3.4.4. Heart Failure (Table 6)

Four studies [19–21, 36] reported heart failure in three different comparisons. Meta-analysis of three studies [19–21] found that CDDP was associated with a statistically significant reduction in the risk of heart failure compared with no intervention with no heterogeneity (RR 0.41; 95%CI 0.22 to 0.75; *n* = 782; *I* ^2^ = 0%). Sensitive analysis, excluding the lower quality study [19], got a similar conclusion (RR 0.30; 95%CI 0.14 to 0.65; two studies, *n* = 282; *I* ^2^ = 0%) [20, 21]. Compared with propranolol on the basis of conventional therapy, CDDP still presented a statistical difference in reducing heart failure (RR 0.26; 95%CI 0.07 to 0.99; one study, *n* = 138) [21]. Nevertheless, compared with placebo on the basis of conventional therapy, CDDP showed no effect in the reduction of heart failure (RR 0.63; 95%CI 0.19 to 2.09; one study, *n* = 63) [36]. The associated risk of bias is presented in Figure 5. And the quality of evidence in the main comparison (CDDP versus no intervention) was moderate (Table 4).

#### 3.4.5. Recurrent Angina (Table 6)

Three studies [19, 20, 36] assessed the number of patients having recurrent angina in two different comparisons. While meta-analysis of two studies showed that CDDP was associated with a statistically significant reduction in the risk of recurrent angina compared with no intervention; the heterogeneity was significant (RR 0.43; 95%CI 0.29 to 0.64; *n* = 636; *I* ^2^ = 61%) [19, 20]. We, hence, examined the data and looked over the papers carefully. We found that besides the types of conventional therapy, the sample sizes between the two studies were also of big differences. Furthermore, one study was high risk of bias [19]. Random effects model, therefore, was used and got a different result without statistical difference (RR 0.33; 95%CI 0.10 to 1.03; *n* = 636). Compared with placebo on the basis of conventional therapy, CDDP still showed no effect in the reduction of recurrent angina (RR 0.55; 95%CI 0.29 to 1.02; one study, *n* = 63) [36].  Figure 6 presents the associated risk of bias. The quality of evidence in the main comparison (CDDP versus no intervention) was low (Table 4).

#### 3.4.6. Readmission (Table 6)

Only one study reported readmission in the comparison of CDDP plus conventional therapy versus placebo plus conventional therapy (RR 0.38; 95%CI 0.09 to 1.52; *n* = 63) [36]. The associated risk of bias is presented in Figure 7.

#### 3.4.7. Quality of Life (Table 7)

One study assessed QOL by questionnaire score. The questionnaire was designed referring to TOMHS and SF-36. Compared with placebo group on the basis of conventional therapy, patients in the group treated with CDDP had higher scores (MD 12.60; 95%CI 3.23 to 21.97; *n* = 63) [36]. The associated risk of bias is presented in Figure 8.

#### 3.4.8. Left Ventricular Ejection Fraction (Table 7)

Five studies [19, 21, 33, 34, 36] assessed LVEF in three different comparisons. Meta-analysis (random effects model) of four studies [19, 21, 33, 34] found that CDDP was associated with a statistically significant increase in LVEF compared with no intervention (MD 4.79%; 95%CI 3.31 to 6.28; *n* = 781). For the significant heterogeneity (*I* ^2^ = 51%) among the studies, we examined the data and looked over the papers carefully. We found that there was a significant difference in the duration of treatment among the studies. Therefore, we conducted a subgroup analysis according to the duration of treatment. In the subgroup analysis of patients with 30 days to six weeks treatment [19, 33] versus six months to 12 months treatment [21, 34], the test effect still had statistical significant but without significant heterogeneity: MD 5.71% (95%CI 4.38 to 7.04; two studies, *n* = 590; *I* ^2^ = 9%) for 30 days to six weeks treatment versus MD 3.82% (95%CI 2.46 to 5.19; two studies, *n* = 191; *I* ^2^ = 22%) for six months to 12 months treatment. Compared with placebo on the basis of conventional therapy, CDDP also presented a statistical difference in the increase of LVEF (MD 5.48%; 95%CI 1.10 to 9.86; one study, *n* = 63) [36]. In addition, a single study reported a similar result between CDDP and propranolol (MD 1.60%; 95%CI 0.05 to 3.15; *n* = 138) [21].  Figure 9 presents the associated risk of bias. The quality of evidence in the main comparison (CDDP versus no intervention) was low (Table 4).

#### 3.4.9. Adverse Events

One of the seven studies reported adverse events [36]. The authors described that there were mild adverse events in the CDDP group such as blushing (1/63 patient), abdominal distention (2/63 patients), dizziness, and distention of head (2/63 patients). However, all of the adverse events remitted spontaneously. There were no significant differences between the two groups in blood glucose, hepatic function, and renal function after treatment. The associated risk of bias is presented in Figure 10.

## 4. Discussion

Seven RCTs including 1215 participants were included in this review. CDDP presented statistically significant benefit on the incidence of cardiac death and heart failure as compared with no intervention based on conventional therapy for AMI. Compared with propranolol, CDDP showed the similar effect on heart failure. In addition, the benefit of CDDP on LVEF was statistically significant both in short-term (30 days to six weeks) and long-term (six months to 12 months) treatment compared with no intervention, placebo, or propranolol. CDDP was also associated with a statistically significant improvement in QOL compared with placebo on the basis of conventional therapy. However, it was not associated with a statistically significant effect on RMI, readmission, or recurrent angina. Unfortunately, no data was available to assess the effect of CDDP on revascularization.

The discrepancy between the effect on all-cause mortality before and after sensitive analysis might be related to the lower quality study [19]. Although CDDP was found to be beneficial for the reduction of all-cause mortality after sensitive analysis, the effect still need to be demonstrated due to the low quality of the evidence.

When we mention TCM, often natural products with fewer side effects come to mind. In fact, systematic reviews [22–24] do indicate fewer mild side effects of CDDP for angina pectoris. A latest parallel double blind randomized placebo-controlled trial also showed no significant adverse effects of CDDP for hypercholesterolemia patients [9]. However, in this review, only one study with small simple size described mild adverse events of CDDP with spontaneous remission. Due to the insufficient data, it is too early to evaluate the safety of CDDP for AMI patients at present. We, therefore, suggest detailed description of adverse events in the future studies of CDDP.

We have to consider a number of limitations in this review before recommending the conclusion to clinical practitioners. (1) We might miss some unpublished relevant studies since we only searched unpublished studies from CPCD, CDFD, and CMFD. What is more, we could not create a funnel plot to check for possible publication bias for each outcome due to the low number of included studies. Publication bias might exist in our results. (2) None of the included studies was assessed to be at low risk of bias. The main reasons are as follows: firstly, the method of random sequence generation was unclear in most of the studies, and only one study reported allocation concealment; most of the studies might have selection bias; secondly, no study described double blind method as well as the blinding of outcome assessment; both selection bias and detection bias might exist in the conclusion; thirdly, neither withdrawals nor losses to follow up was reported in each study; this could lead to a high risk of attrition bias; fourthly, one study [19] had selective reporting on cardiac mortality and RMI which should be reported in accordance with its study plan; this could induce reporting bias. In addition, all of the included studies did not mention ITT analysis, which might lead to some other bias. (3) Most of the durations of follow up were short; the reliability and validity of some outcomes such as mortality could be influenced. (4) The small number of included studies and the different comparisons among the studies precluded us from conducting subgroup analyses to explore effect modifiers such as duration of intervention and type of conventional therapy. (5) For some outcomes, only single study provided data and most of the studies did not meet the calculated optimal information size. This might influence the precision of results, which could downgrade the quality of evidence. (6) We assessed the quality of evidence for each outcome according to the GRADE approach with caution. However, the overall quality of evidence in the main comparison was poor, which can weaken the strength of recommendation.

Although this systematic review suggests some benefits of CDDP for AMI patients, the recommendation of findings was limited due to the poor quality studies. Therefore, rigorously designed clinical trials are warranted to further demonstrate the effectiveness and safety of CDDP for AMI. Moreover, we suggest that researchers of RCTs provide complete, clear, and transparent information on their methodologies and findings in the future. This is important for readers or reviewers to assess and use RCTs accurately. Thus, we expect that more RCTs of TCM will be appropriately designed, conducted, and reported according to the CONSORT statement [37] or the CONSORT statement for herbal interventions [38].

## 5. Conclusion

This systematic review found the following potential benefits from CDDP added to conversional therapy in AMI patients: reduction of cardiac death and heart failure, improvement of QOL and LVEF. However, the benefits should be considered due to the poor quality of evidence. In addition, the safety of CDDP has not been confirmed for the deficiency of available studies. More high quality evidence from high quality RCTs is needed to support the clinical use of CDDP for AMI patients.

## Figures and Tables

**Table 1:** Compositions of compound Danshen dropping pill.

Common name	Pinyin name	Latin name	Pharma. actions
Danshen root	Danshen	*Radix Salviae* *Miltiorrhizae *	Dilates coronary vessels and antimyocardial ischemia inhibit platelet aggregation and thrombosis, decrease cholesterol and endothelial damage, scavenge free radicals, antilipid peroxidative, and antiatherosclerosis, and reduce myocardial ischemia-reperfusion injury, anti-inflammatory [10, 11].

Sanchi root	Sanqi	*Radix Notoginseng *	Dilates blood vessel increases blood platelet number to promote hemostasis, inhibits platelet aggregation and thrombosis, and reduces viscosity of whole blood, decreases the heart rate and myocardial ischemia-reperfusion injury, inhibits proliferation of vascular smooth muscle cell, decreases cholesterol and antiatherosclerosis, antioxidation [12, 13].

Borneol	Bingpian	*Borneolum Syntheticum *	Analgesia and sedation boost other drugs' bioavailability, anti-inflammatory, and decreases the heart rate and myocardial oxygen consumption [14, 15].

**Table 2:** Search strategy for the Cochrane library.

Strategy	
No. 1 Danshen pill	
No. 2 salvia pill	
No. 3 compound Danshen	
No. 4 compound salvia	
No. 5 composite Danshen	
No. 6 composite salvia	
No. 7 Dantonic Pill	
No. 8 CDDP	
No. 9 CSDP	
No. 10 FFDS	
No. 11 myocardial infarction [MeSH]	
No. 12 coronary disease [MeSH]	
No. 13 coronary artery disease [MeSH]	
No. 14 acute coronary syndrome [MeSH]	
No. 15 myocardial infarct∗	
No. 16 AMI	
No. 17 MI	
No. 18 acute coronary syndrome	
No. 19 (1, 2, 3, 4, 5, 6, 7, 8, 9, or 10)	
No. 20 (11, 12, 13, 14, 15, 16, 17, or 18)	
No. 21 (19 and 20)	

**Table 3:** Characteristics of included studies.

ID	Sample size (*I*/*C*)	Age (y, *I*/*C*)	Diagnostic criteria of AMI	Type of AMI	Intervention	Control	Duration of treatment	Follow up	Outcomes	Baseline report
Li et al. 2011 [19]	500 (252/248)	60.10 ± 9.60/ 56.70 ± 7.80	Not specified	STEMI	CDDP 10 pills tid + CT (the same as control)	CT (western medicines + PCI)	30 days	30 days	All-cause mortality, shock, arrhythmia, LVEF%, HF, angina, myocardial enzyme.	Yes
Lin 2011 [33]	90 (46/44)	43–75/36–72	ACC/AHA 2004	Unclear	CDDP 10 pills tid + CT (the same as control)	CT (western medicines)	6 weeks	6 weeks	LVEF%, WBC, CRP, Chinese symptoms.	Yes (narrative only)
Ma 2010 [35]	163 (78/85)	62.55 ± 11.95/ 66.02 ± 11.40	Not specified	STEMI	CDDP 10 pills tid + CT (the same as control)	CT (western medicines or plus PCI/thrombolysis)	1 month	3 months	All-cause mortality, IL-6, cardiac mortality, hs-CRP, MACEs, MMP-9, TNF-a.	Yes
Li et al. 2010 [36]	63 (42/21)	58.40 ± 11.60	Not specified	Unclear	CDDP 10 pills tid + CT (the same as control)	Placebo + CT (western Medicines + thrombolysis).	4 months	4 months	Cardiac mortality, LVEF%, readmission, QOL, RMI, HF, angina, adverse events.	Yes (narrative only)
Guo et al. 2010 [20]	136 (76/60)	55.60 ± 12.5/ 51.80 ± 13.60	WHO	Unclear	CDDP 10 pills tid + CT (the same as control)	CT (western medicines + thrombolysis)	4 weeks	4 weeks	All-cause mortality, shock, HF, recanalization, angina, myocardial enzyme.	Yes (narrative only)
Xu and Wang 2007 [21]	218 (66/72/80)	36–75/37–78 /32–79	WHO	With Q-wave	CDDP 10 pills tid d1-60, then 5 pills tid + CT (the same as control)	Propranolol 10~15 mg tid + CT (no detail); CT (no detail)	12 months	12 months	All-cause mortality, RMI, cardiac mortality, HF, arrhythmia, LVEF%.	Yes (narrative only)
Mei et al. 2006 [34]	45 (23/22)	56.11 ± 11.13	Not specified	Unclear	CDDP 10 pills tid + CT (the same as control)	CT (western medicines)	6 months	6 months	LVEF%, SV, CO	Unclear

Notes: CT: conventional therapy; HF: heart failure; AHA: American heart Association; ACC: American College of Cardiology; CRP: C-reactive protein; hs-CRP: high sensitive C-reactive protein; WBC: white blood cell; MACEs: major adverse cardiac events; WHO: World Health Organization; TNF-*α*: tumor necrosis factor-alpha; MMP-9: matrix metalloproteinase-9; IL-6: interleukin-6; SV: stroke volume; CO: cardiac output; tid: three times a day.

**Table 4:** GRADE analysis: summary of findings for the main comparison.

Compound Danshen dropping pill versus no intervention for acute myocardial infarction						
Patient or population: patients with acute myocardial infarction						
Settings: inpatients and outpatients						
Intervention: compound Danshen dropping pill (CDDP)						

Outcomes	Illustrative comparative risks* (95% CI)		Relative effect (95% CI)	None of Participants (studies)	Quality of the evidence (GRADE)	Comments
	Assumed risk	Corresponding risk				
	Control	CDDP				

*All-cause mortality* Follow-up: 4 weeks–12 months	*Study population *		RR 0.65 (0.37–1.14)	945 (4 studies)	*⊕⊕* *⊝* *⊝* low^1,2^	
	63 per 1000	41 per 1000 (23–72)				
	*Moderate *				
	104 per 1000	68 per 1000 (38–119)			

*Cardiac mortality* Follow-up: 3–12 months	*Study population *		RR 0.43 (0.2–0.95)	309 (2 studies)	*⊕⊕* *⊕* *⊝* moderate^3,4^	
	127 per 1000	55 per 1000 (25–121)				
	*Moderate *				
	127 per 1000	55 per 1000 (25–121)			

*Recurrent myocardial infarction* Follow-up: mean 12 months	*Study population *		RR 0.30 (0.07–1.38)	146 (1 study)	*⊕⊕* *⊝* *⊝* low^5^	
	100 per 1000	30 per 1000 (7–138)				
	*Moderate *				
	100 per 1000	30 per 1000 (7–138)			

*Heart failure* Follow-up: 4 weeks–12 months	*Study population *		RR 0.41 (0.22–0.75)	782 (3 studies)	*⊕⊕* *⊕* *⊝* moderate^1^	
	85 per 1000	35 per 1000 (19–64)				
	*Moderate *				
	150 per 1000	62 per 1000 (33–112)		

*Recurrent angina* Clinical diagnosis based on patients complaint Follow-up: 4 weeks–30 days	*Study population *		RR 0.33 (0.1–1.03)	636 (2 studies)	*⊕⊕* *⊝* *⊝* low^1,6,7^	
	211 per 1000	70 per 1000 (21–217)				
	*Moderate *				
	201 per 1000	66 per 1000 (20–207)			

*Left ventricular ejection fraction* Measured with echocardiogram. Scale from 30% to 75%. Duration of treatment: 4–6 weeks	The mean left ventricular ejection fraction in the control groups was 50.48%^9^	The mean left ventricular ejection fraction in the intervention groups was 5.71% higher (4.38%–7.04% higher)		590 (2 studies)	*⊕⊕* *⊝* *⊝* low^1,8^	Higher score indicates improvement

*Left ventricular ejection fraction* Measured with echocardiogram. Scale from: 30%–75%. Duration of treatment: 6–12 months	The mean left ventricular ejection fraction in the control groups was 49.71%^9^	The mean left ventricular ejection fraction in the intervention groups was 3.82% higher (2.46%–5.19% higher)		191 (2 studies)	*⊕⊕* *⊝* *⊝* low^3,8^	Higher score indicates improvement

*The basis for the assumed risk (e.g., the median control group risk across studies) is provided in footnotes. The corresponding risk (and its 95% confidence interval) is based on the assumed risk in the comparison group and the relative effect of the intervention (and its 95% CI).

CI: confidence interval; RR: risk ratio; GRADE Working Group grades of evidence:

High quality: further research is very unlikely to change our confidence in the estimate of effect.

Moderate quality**:** further research is likely to have an important impact on our confidence in the estimate of effect and may change the estimate.

Low quality: further research is very likely to have an important impact on our confidence in the estimate of effect and is likely to change the estimate.

Very low quality: we are very uncertain about the estimate.

^1^One study had selective reporting. For the other studies, the overall risk of bias was felt to be unclear.

^2^95%CI includes possibility of both benefits and harms, and the sample size was not the optimal information size. After sensitive analysis excluding the lower quality study, the result suggested benefit, but the sample size was still small.

^3^The overall risk of bias of the studies was unclear. The sample size was not the optimal information size.

^4^95%CI included only benefit, so we were cautious about downgrading the imprecision although the sample size was less than the optimal information size.

^5^Unclear risk of bias and only 146 patients enrolled. 95%CI included possibility of both benefits and harms.

^6^The heterogeneity (*I* ^2^ = 61%) can be explained by the major differences of conventional therapy and sample size between the two studies, and this outcome is not so important to affect the decision-making; therefore, we did not downgrade for this factor.

^7^95%CI suggested benefit as well as no benefit.

^8^This is an indirect outcome for AMI patients.

^9^Final measurements at the end of the study.

**Table 5:** Analyses of primary outcomes.

Outcomes (comparisons)	Treatment (*n*/*N*)	Control (*n*/*N*)	Weight (%)	RR	95% CI
*(1) All-cause mortality *					
(1.1) CDDP + conventional therapy versus conventional therapy					
Guo et al. 2010 [20]	5/76	5/60	19.10	0.79	[0.24, 2.60]
Li et al. 2011 [19]	6/252	4/248	13.80	1.48	[0.42, 5.17]
Ma 2010 [35]	5/78	11/85	36.10	0.50	[0.18, 1.36]
Xu and Wang 2007 [21]	3/66	10/80	31.00	0.36	[0.10, 1.27]

Total (FEM, *I* ^2^ = 0%)			100.00	0.65	[0.37, 1.14]

*Sensitive analysis *					
Guo et al. 2010 [20]	5/76	5/60	22.20	0.79	[0.24, 2.60]
Ma 2010 [35]	5/78	11/85	41.80	0.50	[0.18, 1.36]
Xu and Wang 2007 [21]	3/66	10/80	35.90	0.36	[0.10, 1.27]

**Total (FEM, ** **I** ^2^ **= 0%)**			**100.00**	**0.51 **	[**0.27, 0.98**]

(1.2) CDDP + conventional therapy versus propranolol + conventional therapy					
Xu and Wang 2007 [21]	3/66	5/72	100.00	0.65	[0.16, 2.63]
*(2) Cardiac mortality *					
(2.1) CDDP + conventional therapy versus conventional therapy					
Ma 2010 [35]	5/78	11/85	53.80	0.50	[0.18, 1.36]
Xu and Wang 2007 [21]	3/66	10/80	46.20	0.36	[0.10, 1.27]

**Total (FEM, ** **I** ^2^ **= 0%)**			**100.00**	**0.43 **	[**0.20, 0.95**]

(2.2) CDDP + conventional therapy versus placebo + conventional therapy					
Li et al. 2010 [36]	1/42	1/21	100.00	0.50	[0.03, 7.60]
(2.3) CDDP + conventional therapy versus propranolol + conventional therapy					
Xu and Wang 2007 [21]	3/66	4/72	100.00	0.81	[0.17, 3.76]
*(3) Recurrent myocardial infarction *					
(3.1) CDDP + conventional therapy versus conventional therapy					
Xu and Wang 2007 [21]	2/66	8/80	100.00	0.30	[0.07, 1.38]
(3.2) CDDP + conventional therapy versus placebo + conventional therapy					
Li et al. 2010 [36]	3/42	3/21	100.00	0.50	[0.11, 2.27]
(3.3) CDDP + conventional therapy versus propranolol + conventional therapy					
Xu and Wang 2007 [21]	2/66	3/72	100.00	0.73	[0.13, 4.22]

**Table 6:** Analyses of secondary outcomes.

Outcomes (comparisons)	Treatment (*n*/*N*)	Control (*n*/*N*)	Weight (%)	RR	95% CI
*(1) Heart failure *					
(1.1) CDDP + conventional therapy versus conventional therapy					
Xu and Wang 2007 [21]	3/66	12/80	32.40	0.30	[0.09, 1.03]
Guo et al. 2010 [20]	5/76	13/60	43.40	0.30	[0.11, 0.80]
Li et al. 2011 [19]	6/252	8/248	24.10	0.74	[0.26, 2.10]

**Total (FEM, ** **I** ^2^ **= 0%)**			**100.00**	**0.41**	[**0.22, 0.75**]

Sensitive analysis					
Xu and Wang 2007 [21]	3/66	12/80	57.30	0.30	[0.09, 1.03]
Guo et al. 2010 [20]	5/76	13/60	42.70	0.30	[0.11, 0.80]

**Total (FEM, ** **I** ^2^ **= 0%)**			**100.00**	**0.30 **	[**0.14, 0.65**]

(1.2) CDDP + conventional therapy versus placebo + conventional therapy					
Li et al. 2010 [36]	5/42	4/21	100.00	0.63	[0.19, 2.09]
(1.3) CDDP + conventional therapy versus propranolol + conventional therapy					
** Xu and Wang 2007 [21]**	**3/66**	**11/72**	**100.00**	**0.26 **	[**0.07, 0.99**]
*(2) Recurrent angina *					
(2.1) CDDP + conventional therapy versus conventional therapy					
Guo 2010 [20]	2/76	11/60	33.40	0.14	[0.03, 0.62]
Li et al. 2011 [19]	27/252	54/248	66.60	0.49	[0.32, 0.75]

Total (REM, *I* ^2^ = 61%)			100.00	0.33	[0.10, 1.03]

(2.2) CDDP + conventional therapy versus placebo + conventional therapy					
Li et al. 2010 [36]	12/42	11/21	100.00	0.55	[0.29, 1.02]
*(3) Readmission *					
CDDP + conventional therapy versus placebo + conventional therapy					
Li et al. 2010 [36]	3/42	4/21	100.00	0.38	[0.09, 1.52]

**Table 7:** Analyses of secondary outcomes.

Outcomes (comparisons)	Treatment			Control			Weight (%)	MD	95% CI
	Mean	SD	*N*	Mean	SD	*N*			
*(4) LVEF% *									
(4.1) CDDP + conventional therapy versus conventional therapy									
Mei et al. 2006 [34]	60.80	7.20	23	59.20	6.80	22	10.50	1.60	[−2.49, 5.69]
Xu and Wang 2007 [21]	51.20	4.30	66	47.10	4.60	80	34.60	4.10	[2.65, 5.55]
Li et al. 2011 [19]	57.10	8.70	252	51.90	9.90	248	31.70	5.20	[3.57, 6.83]
Lin 2011 [33]	54.50	6.80	46	47.80	3.90	44	23.30	6.70	[4.42, 8.98]

**Total (REM, ** **I** ^2^ **= 51%)**							**100.00 **	**4.79 **	[**3.31, 6.28**]

*Subgroup analysis (according to duration of treatment) *									
(4.1.1) 30 days–6 weeks									
Li et al. 2011 [19]	57.10	8.70	252	51.90	9.90	248	33.90	5.20	[3.57, 6.83]
Lin 2011 [33]	54.50	6.80	46	47.80	3.90	44	17.40	6.70	[4.42, 8.98]

**Subtotal (FEM, ** **I** ^2^ **= 9%)**							**51.30 **	**5.71 **	[**4.38, 7.04**]

(4.1.2) 6 months–12 months									
Mei et al. 2006 [34]	60.80	7.20	23	59.20	6.80	22	5.40	1.60	[−2.49, 5.69]
Xu and Wang 2007 [21]	51.20	4.30	66	47.10	4.60	80	43.30	4.10	[2.65, 5.55]

**Subtotal (FEM, ** **I** ^2^ **= 22%)**							**48.70 **	**3.82 **	[**2.46, 5.19**]

(4.2) CDDP + conventional therapy versus placebo + conventional therapy									
Li et al. 2010 [36]	55.69	9.34	42	50.21	7.83	21	100.00	5.48	[1.10, 9.86]
(4.3) CDDP + conventional therapy versus propranolol + conventional therapy									
Xu and Wang 2007 [21]	51.20	4.30	66	49.60	5.00	72	100.00	1.60	[0.05, 3.15]
*(5) Quality of life (score) *									
CDDP + conventional therapy versus placebo + conventional therapy									
Li et al. 2010 [36]	110.28	19.33	42	97.68	17.13	21	100.00	12.60	[3.23, 21.97]
